# Cornwall as a Winter Resort

**Published:** 1890-02-22

**Authors:** 


### CLIMATOLOGY.

### CORNWALL AS A WINTER RESORT.

We have heard a good deal latterly about Cornwall as a winter resort, and a pamphlet has been published anonymously setting forth the advantages of this part of the country. It is the object of the paper, in the interests of invalids who cannot go abroad, to draw attention to the advantages offered by Cornwall, which, at the cost of a short land journey in direct connexion with the Great Western Railway system, gives the invalid a choice of climates rivalling those of the South of France in mildness, while surpassing them in geniality and equability. It appears that more than fifty years ago Dr. Paris and others wrote of the advantages of the Cornwall climate; but owing, it may be presumed, to the extension of facilities for proceeding abroad, the Cornwall climate has not hitherto attracted the number of invalids according to its deserts. This is considered absurd, seeing that it is only nine and a-quarter hours from London. The advantages offered are the mildest climate in the kingdom in winter, and the most oxygenated air in summer. January at Penzance is as warm as at Madrid, Florence, and Constantinople, and July is as cool as at St. Petersburgh. And the reason is the Gulf Stream. The myrtle, hydrangea, and fuchsia live and thrive throughout the winter. Snow, which rarely falls, is very evanescent, and ice is seldom thick enough for the skater. Crocuses and snowdrops are seen before they have pierced the snows of Parma, and in this particular Cornwall compares favourably with Naples. Figures are given showing the winter mean range of temperature at Penzance, which is 4·64 deg.; while at Cannes it is 10·7 deg.; at Mentone, 9·6 deg.; at Nice, 11 deg.; at Pau, 10 6 deg. There are, however, differences in the local climates of Cornwall, chiefly depending on elevation. And the pamphlet under notice gives an account of Falmouth, Penzance, St. Ives, Newquay, and the Scilly Isles. All is painted *coleur de rose*, and is certainly calculated to cause the incautious invalid to proceed to Cornwall at once. Especially as it is undoubtedly all truth which is stated. But there are sins of omission as well as of commission. Although the pamphlet bristles with tables of temperature, and with lists of exotic flowers, there is no information given as to these important points—rainfall and humidity. Indeed, so far as we can see, the word rainfall is only once mentioned, where it is stated that "the high land on the central ridge of the country is much colder in winter, and much hotter in summer, and has a much heavier rainfall than Cornwall generally." Now, taking Penzance as a standard, we find that the rainfall is large—about 44 inches annually, or almost twice as much as London—and there are about 179 rainy days in the year. Then, again, we are not told much in this Cornwall pamphlet about winds, which are usually southerly. The climate of Cornwall may, therefore, be regarded as warm, moist, mild, and relaxing. Other disadvantages are exposure to sudden storms of wind and rain, and a humid atmosphere dependent on the constantly recurring rainfall brought by the prevalence of southerly winds. Late and comparatively cold springs are also unfavourable. It is a peculiarity of the climate of the whole of Cornwall that the spring is less warm than at other places as compared with the difference between the other seasons; the variation being, in fact, very little at this time between Penzance and London. Now we do think that when a pamphlet of the kind is written the disadvantages, as well as the advantages, should be fairly stated.

